# VennDiagram: a package for the generation of highly-customizable Venn and Euler diagrams in R

**DOI:** 10.1186/1471-2105-12-35

**Published:** 2011-01-26

**Authors:** Hanbo Chen, Paul C Boutros

**Affiliations:** 1 Informatics and Biocomputing Platform, Ontario Institute for Cancer Research, MaRS Centre, South Tower, 101 College Street, Suite 800, Toronto, Ontario, M5G 0A3, Canada

## Abstract

**Background:**

Visualization of orthogonal (disjoint) or overlapping datasets is a common task in bioinformatics. Few tools exist to automate the generation of extensively-customizable, high-resolution Venn and Euler diagrams in the R statistical environment. To fill this gap we introduce *VennDiagram*, an R package that enables the automated generation of highly-customizable, high-resolution Venn diagrams with up to four sets and Euler diagrams with up to three sets.

**Results:**

The *VennDiagram *package offers the user the ability to customize essentially all aspects of the generated diagrams, including font sizes, label styles and locations, and the overall rotation of the diagram. We have implemented scaled Venn and Euler diagrams, which increase graphical accuracy and visual appeal. Diagrams are generated as high-definition TIFF files, simplifying the process of creating publication-quality figures and easing integration with established analysis pipelines.

**Conclusions:**

The *VennDiagram *package allows the creation of high quality Venn and Euler diagrams in the R statistical environment.

## Background

The visualization of complex datasets is an increasingly important part of biology. Many experiments involve the integration of multiple datasets to understand complementary aspects of biology. These overlapping results can be visualized in a number of ways, including textual tables (e.g. two-way tables), network diagrams [1,2] and in some cases heatmaps [3,4]. Venn diagrams have seen increasing use due to their familiarity, ease-of-interpretation, and graphical simplicity. For the purpose of this publication, Venn diagrams can be defined as diagrams that use simple geometrical shapes such as circles and ellipses to display all 2^n^-1 possible areas created by the interaction of n sets. The use of simple geometrical shapes reduces figure complexity and size relative to space-consuming tables or network layouts.

However, despite this popularity, there are currently few packages for generating Venn diagrams in the widely-used R statistical environment. These packages are limited in their ability to generate high-resolution, publication-quality Venn diagrams in that they allow little customization of colours, line-types, label-placement, and label font. Numerous special-cases are handled inappropriately, and the output is not usually in the format of high-resolution, publication-quality TIFF files. Other, non-R-based local or web-based software capable of generating Venn diagrams exist, such as Venny [5], BioVenn [6], ConSet [7], and VennMaster [8]. All of these suffer from some of the weaknesses listed above. Further, integration into standard R-based statistical/computational pipelines such as the widely used BioConductor libraries of the R statistical environment [9] is viable, but not technically trivial.

Additionally, if some intersecting or non-intersecting areas in a Venn diagram do not exist, another class of diagrams called Euler diagrams may be more desirable. Euler diagrams are equivalent to Venn diagrams when all intersecting and non-intersecting areas exist. However, areas containing zero elements are shown on Venn diagrams (by definition), whereas Euler diagrams show only non-zero areas. In many cases, Euler diagrams further reduce figure complexity, increase graphical accuracy and improve overall readability relative to Venn diagrams. Unfortunately, almost all existing packages cannot generate publication-quality Euler diagrams in R, although VennEuler does generate Euler diagrams.

To address these issues we introduce *VennDiagram*, an R package for generating highly customizable, high-resolution Venn diagrams with up to four sets and Euler diagrams of two or three sets in the R statistical environment.

## Implementation

The *VennDiagram *package has been developed in and designed for the R statistical environment. The R environment is open-source and available online under the GNU General Public License (GPLv2). R was chosen because of its open-source nature, versatile functions, and general preference within the bioinformatics community. The use of R should facilitate integration with existing data-analysis pipelines. All code was designed and tested using version 2.12.1 (32-bit and 64-bit versions) of R. The VennDiagram package is available as Additional Files 1 (linux .tar.gz file) and 2 (windows .zip file).

*VennDiagram *uses the grid package for graphics. The grid package is a base (standard) package available in all installations, and offers more manoeuvrability than default R graphics in terms of graphical options and the existence of modifiable grid objects. *VennDiagram *uses these features to dynamically stretch/compress diagrams to fit the dimensions of the output file and to offer a vast number of graphical options.

## Results

**Figure 1:**
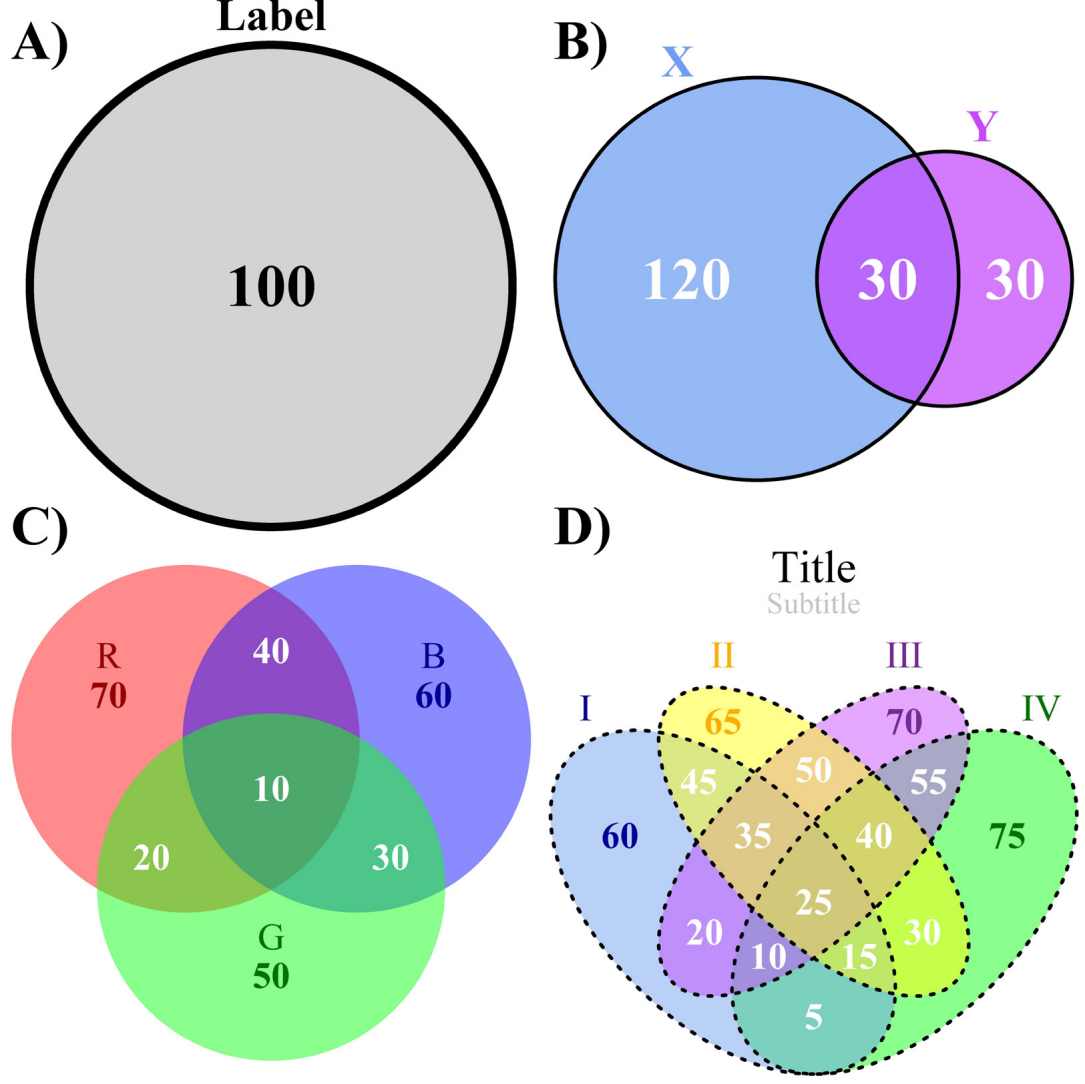
**The four types of Venn diagrams drawn by the *VennDiagram *package**. A) A one-set Venn diagram showing rudimentary customizable features such as label font size, label font face, and shape-fill. B) A two-set Venn diagram showing more advanced features such as scaling, individual shape-fill specifications, and individual caption label placement. C) A three-set Venn diagram showing a different shape-line type ("transparent") and the "text" option of caption label placement where the caption labels are attached to area labels. D) A four-set Venn diagram showing a combination of all previous features plus the ability to customize titles. The code to generate all diagrams shown here is included in Additional File 3.

Almost all graphical options in the *VennDiagram *package have been parameterized and made customizable. Default values were selected to generate sensible diagrams, so in simple cases a high level of customization is not required. Figure 1 highlights the diversity of parameterizations available. Four major graphical parameter groups exist: shape-fill, shape-line, labels and titles. Shape-fill refers to the colours within each circle or ellipse. All colours available in the R environment can be used, and alpha-blending can be adjusted on a per-shape basis. Shape-lines are the lines that surround each circle or ellipse. They can be entirely absent (figure 1C), solid (figures 1A and 1B), or any other R line-type available (figure 1D). Their colour can be changed, and each shape can have a separate set of parameters. Labels refer both to the captions describing each circle or ellipse and to the numbers within them. Again, these can be customized in terms of colour, font-type, and font-size with any available R parameter. The positions of caption labels can also be customized. Titles, which include the main title and the subtitle as demonstrated in figure 1D, can also be customized in the same way as labels.

**Figure 2:**
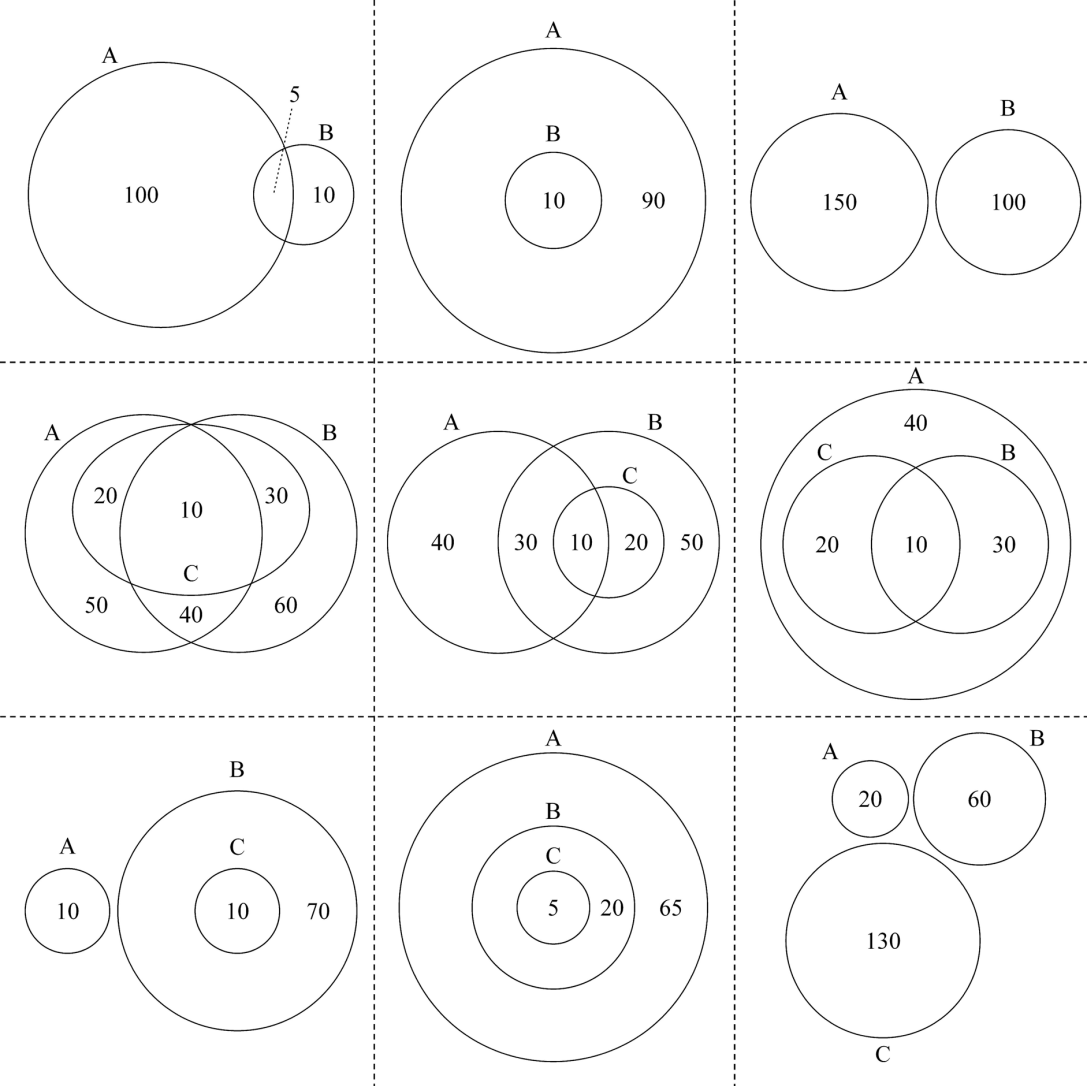
**Selected Venn diagram special cases and Euler diagrams drawn by the *VennDiagram *package**. Row 1, column 1: automatically drawn, customizable lines that optimize display of partial areas when individual partial areas become too small in two-set Venn diagrams. Row 1, column 2: a two-set Euler diagram showing total inclusion of one of the sets. Row 1, column 3: a two-set Euler diagram showing two distinct sets. Row 2, column 1: a three-set Euler diagram where one set has no discrete elements. Row 2, column 2: a three-set Euler diagram where one set has no discrete elements is totally included in one of the other two sets. Row 2, column 3: a three-set Euler diagram where two sets have no discrete elements and are included in a larger third set. Row 3, column 1: a three-set Euler diagram showing total inclusion of two sets that are distinct from the third set. Row 3, column 2: a three-set Euler diagram where one set is totally included in another set, which is itself totally included in the third set. Row 3, column 2: a three-set Euler diagram showing three distinct sets. The code to generate all diagrams shown here is included in Additional File 3.

Beyond these specific graphic elements, *VennDiagram *also offers many general options, such as the scaling, rotation, or inversion of diagrams. Diagram scaling was implemented with the goal of displaying Venn diagrams where the graphical sizes of the partial areas (areas bound on all sides by curves and that cannot be further subdivided) actually correspond to the numerical values of the number of elements within each region. Scaling of two-set Venn diagrams and a select number of three-set Venn diagrams is possible - we discuss below the challenges of making this possible for all three-set cases. The use of scaling can sometimes lead to overlapping areas being too small for numeric labels. Figure 2, row 1, column 1 shows a programmatically generated connecting line that allows special handling of this case. Automatic recognition of a large number of Euler diagrams is implemented, but this mode can be deactivated with a function-call parameter to plot standard Venn diagrams instead. Figure 2, row 1, columns 2 and 3 show two examples of two-set Euler diagrams. Rows 2 and 3 show a subset of implemented three-set Euler diagrams with row 3 showing those of the scalable variety. We note that figure 2 is presented in black and white to highlight compositional differences across diagrams, but the graphical parameters and customizations used in figure 1 are fully available. While VennDiagram defaults to writing graphics to high-resolution TIFF files, if the filename parameter is set to NULL the raw grid object can be returned and used in any graphics mode available in R. The code to generate all figures is given in Additional File 3 and an example of all available parameterizations is shown in Additional File 4.

### Discussion

During development of the *VennDiagram *package, it was discovered that it was impossible to draw accurate, scaled Venn diagrams with three sets using circles. This conundrum is illustrated in the following scenario. In a system of two circles A and B, the distances between the centres of the circles, d_AB_, could be determined as long as the areas (A_A _and A_B _respectively) and the intersection area (A_A _∩ A_B_) are both known. This is possible because in a two-circle system a single A_A _∩ A_B _corresponds to a unique value for d_AB_. Therefore, a system of three circles A, B, and C, d_AB_, d_BC_, d_AC _could be calculated as long as A_A_, A_B_, A_C_, A_A _∩ A_B_, A_A _∩ A_C_, A_B _∩ A_C _are all known. However, d_AB_, d_BC_, d_AC _make a unique triangle, implying that a Venn diagram can be drawn without ever knowing the overall intersection A_A _∩ A_B _∩ A_C_. In other words, the size of the overlap between all three circles does not alter the presentation of scaled Venn diagrams -- the area is unchanged even if one system has zero overall intersection (i.e. A_A _∩ A_B _∩ A_C _= 0)! This conundrum results from the (arbitrary) choice of circles to represent set size, which reduces the degrees of freedom by one. Unique solutions can be identified by using ellipses or polygons to draw Venn diagrams but the resulting diagrams would lose the instant recognisability and familiarity associated with circular Venn diagrams, defeating the point of a convenient display of information. Non-circular diagrams would also require iterative algorithms to compute the positions and sizes of the shapes, greatly increasing computational burdens, as has been discussed by others [10]. Consequently, scaling of three-set Venn diagrams is disabled in the *VennDiagram *package. Similarly, Venn diagrams containing more than four sets [11,12] were not implemented in the *VennDiagram *package because they become too complex for intuitive visualization.

**Figure 3:**
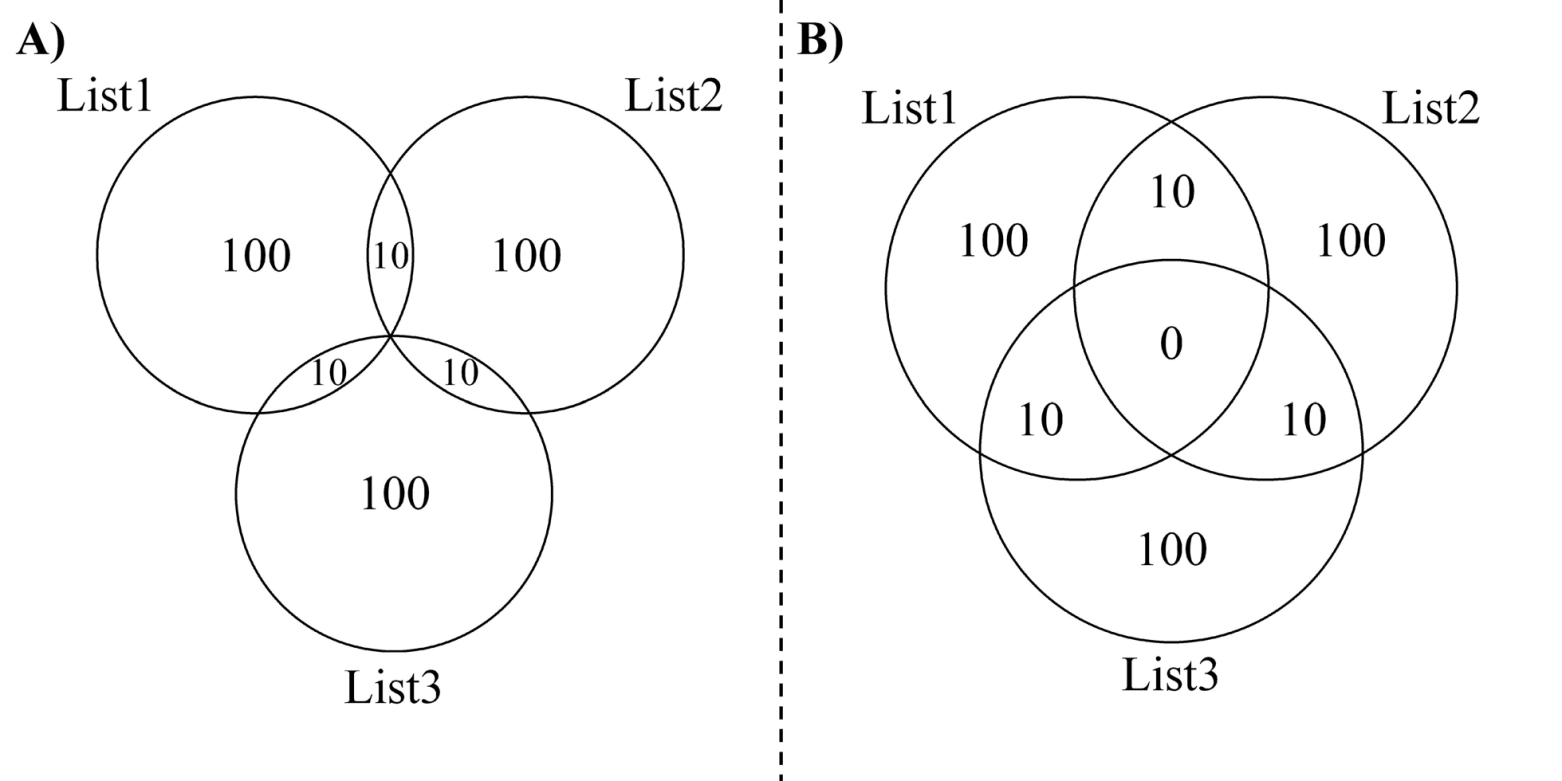
**A side-by-side comparison of an Euler diagram and a Venn diagram for the same hypothetical sets**. A) The Euler diagram shows only non-zero areas and can therefore be more graphically accurate. B) The Venn diagram shows the non-existent area as an area with zero content. Though this is not graphically accurate, it preserves the recognisability of a Venn diagram.

A general caveat when using Euler diagrams is that although they reduce the graphical complexity of some Venn diagrams, their non-traditional shapes may also be less recognizable in some cases. When empty areas are present, the user needs to choose between the familiarity of Venn diagrams and the increased accuracy of Euler diagrams. Figure 3 illustrates a situation where either a Venn or an Euler diagram may be appropriate depending on user preferences.

The *VennDiagram *package handles all two-set Euler diagrams and the majority of all conceivable three-set Euler diagrams. Three-set Euler diagrams that could not be drawn using circles or ellipses are not supported. For example, an Euler diagram for the case where two non-intersecting sets comprise the third set cannot be drawn using circles and ellipses, though it may be drawn using polygons. This type of figure lacks a ready analytical layout and would require iterative fitting; no polygon-requiring Euler diagrams are available, but standard Venn diagrams are available for these few unsupported cases.

After comparing with other programs capable of generating Venn diagrams (Table 1), advantages of the *VennDiagram *package include:

• Drawing Euler diagrams using circles and/or ellipses with two or three sets

• Offering greater customizability to generate more elegant diagrams

• Availability in the widely-used R statistical environment

• Generating high resolution TIFF files that are standard in publications

**Table 1:** A comparison of the features of various programs capable of generating Venn diagrams.

	DrawVenn	Venny	gplots::venn	venneuler	limma::vennDiagram	Google Chart	GeneVenn	VennMaster	BioVenn	VennDiagram
Shape-fill										

Colour				X		X	X	X	X	X

Shape-line										

Style										X

Width					X					X

Colour										X

Caption labels										

Content				X			X	X		X

Colour									X	X

Font							X		X	X

Size							X		X	X

Style										X

Location								X	X (SVG only)	X

Position								X	X (SVG only)	X

Distance								X	X (SVG only)	X

Justification										X

Area labels										

Colour					X				X	X

Font		X					X		X	X

Size		X			X		X		X	X

Style										X

Titles										

Main title						X	X		X	X

Subtitle									X	X

Position									X (SVG only)	X

Colour						X			X	X

Font									X	X

Size						X			X	X

Style										X

Justification										X

Background-fill										

Colour						X			X	

Style						X				

File options										

Output type	None	PNG	R graphics	R graphics	R graphics	PNG/GIF	PNG	SVG/JPEG	SVG/PNG	TIFF/PNG/JPEG/BMP/others

Figure resolution								X	X	X

Data processing										

Built-in gene ID recognition								X	X	

Figure from file(s)							X	X	X	

Specific optimizations								Gene Ontology		

General										

Environment	Java	Web	R	R	R	Web	Web	Java	Web	R

Input format	Direct (slider)	Lists	Lists	Partial areas	R object	Partial areas	Lists	Lists/GoMiner output	Lists	Lists

Maximum sets	3	4	5	3	3	3	3	>5	3	4

Shapes used	Circles/Rectangles	Circles/Ellipses	Circles/Ellipses	Circles	Circles	Circles	Circles	Polygons	Circles	Circles/Ellipses

Scaling	X			X*		X*		X (iterative)	X*	X (2-set only)

Euler diagrams				X				X	X	X

Margin size					X	X				X

Rotation										X

Two-set external lines										X

Other set-specific parameters								X		X

* uses inaccurate 3-set scaling with circles

This table highlights the improvements that the VennDiagram package possesses over other notable Venn diagram-generating software. The highly customizable nature of the VennDiagram package is evident.

## Conclusions

The *VennDiagram *package advances both the ease-of-use and the degree of customizability in the generation of Venn diagrams in a bioinformatics context. While other tools offer much of the functionality presented here, the implementation of all features together in the widely-used R statistical environment will promote the usage of automatically generated Venn diagrams within computational pipelines.

## Availability and Requirements

The *VennDiagram *package itself is available as Additional Files 1 and 2, and will be submitted to CRAN - a global repository of R packages. *VennDiagram *requires R (>2.12.1) and the grid package for R.

## Authors' contributions

HC and PCB conceived of the project. HC wrote the software, which HC and PCB tested and debugged. HC wrote the first draft of the manuscript, which all authors revised and approved.

## Supplementary Material

Additional file 1 The *VennDiagram *R package as a linux-compatible .tar.gz file Click here for file

Additional file 2 The *VennDiagram *R package as a windows-compatible .zip file Click here for file

Additional file 3 Code to generate all Venn diagrams in Figures 1 and 2. Click here for file

Additional file 4 Illustration of the parameters available in *VennDiagram*. Click here for file
