# Supplementary material for: VennDiagram: a package for the generation of highly-customizable Venn and Euler diagrams in R
Source: BMC Bioinformatics. 2011 Jan 26;12:35. doi: 10.1186/1471-2105-12-35 (PMC3041657; doi:10.1186/1471-2105-12-35)
Supplement: Additional file 2 — The VennDiagram R package as a windows-compatible .zip file [file 1471-2105-12-35-S2.ZIP › VennDiagram/html/00Index.html]

R: Generate high-resolution Venn and Euler plots

# Generate high-resolution Venn and Euler plots


---

## Documentation for package ‘VennDiagram’ version 1.0.0

- DESCRIPTION file.

## Help Pages

|  |  |
| --- | --- |
| VennDiagram-package | Venn diagram plotting |
| venn.diagram | Make a Venn Diagram |
| VennDiagram | Venn diagram plotting |
