# Supplementary material for: VennDiagram: a package for the generation of highly-customizable Venn and Euler diagrams in R
Source: BMC Bioinformatics. 2011 Jan 26;12:35. doi: 10.1186/1471-2105-12-35 (PMC3041657; doi:10.1186/1471-2105-12-35)
Supplement: Additional file 4 — Illustration of the parameters available in VennDiagram. [file 1471-2105-12-35-S4.PPT]

## Slide 1
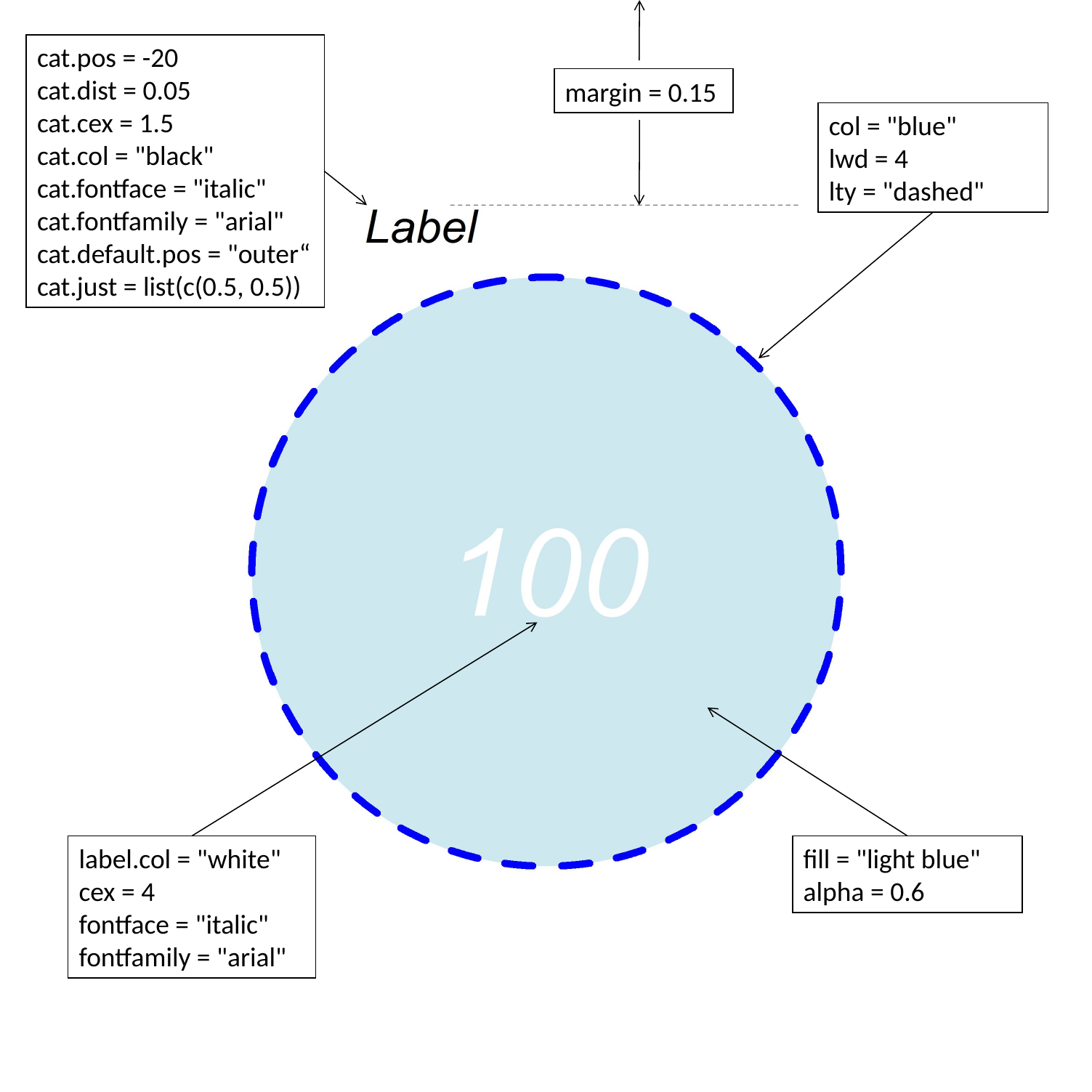

cat.pos = -20
cat.dist = 0.05
cat.cex = 1.5
cat.col = "black"
cat.fontface = "italic"
cat.fontfamily = "arial"
cat.default.pos = "outer“
cat.just = list(c(0.5, 0.5))
margin = 0.15
col = "blue"
lwd = 4
lty = "dashed"
label.col = "white"
cex = 4
fontface = "italic"
fontfamily = "arial"
fill = "light blue"
alpha = 0.6

## Slide 2
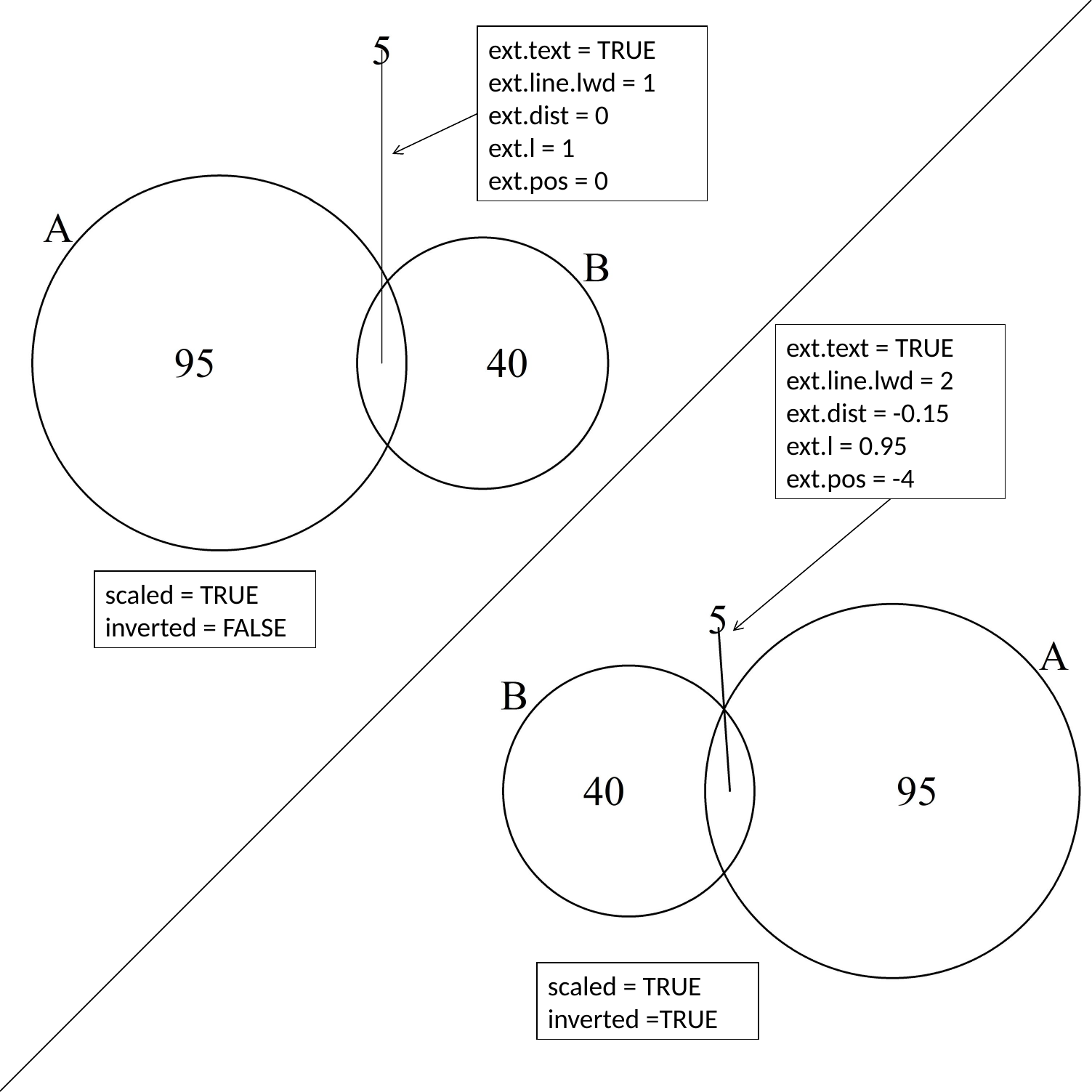

ext.text = TRUE
ext.line.lwd = 1
ext.dist = 0
ext.l = 1
ext.pos = 0
ext.text = TRUE
ext.line.lwd = 2
ext.dist = -0.15
ext.l = 0.95
ext.pos = -4
scaled = TRUE
inverted = FALSE
scaled = TRUE
inverted =TRUE

## Slide 3
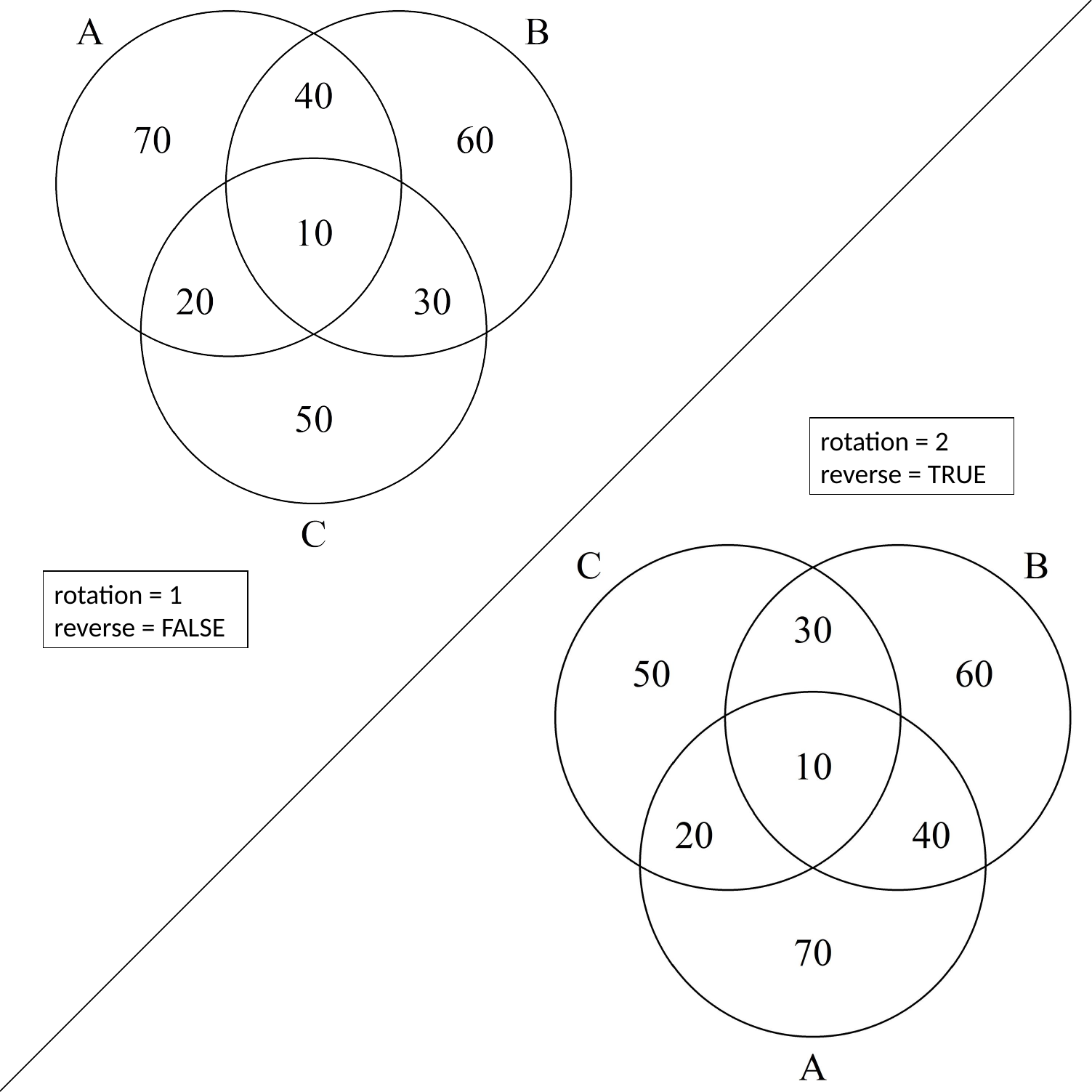

rotation = 2
reverse = TRUE
rotation = 1
reverse = FALSE

## Slide 4
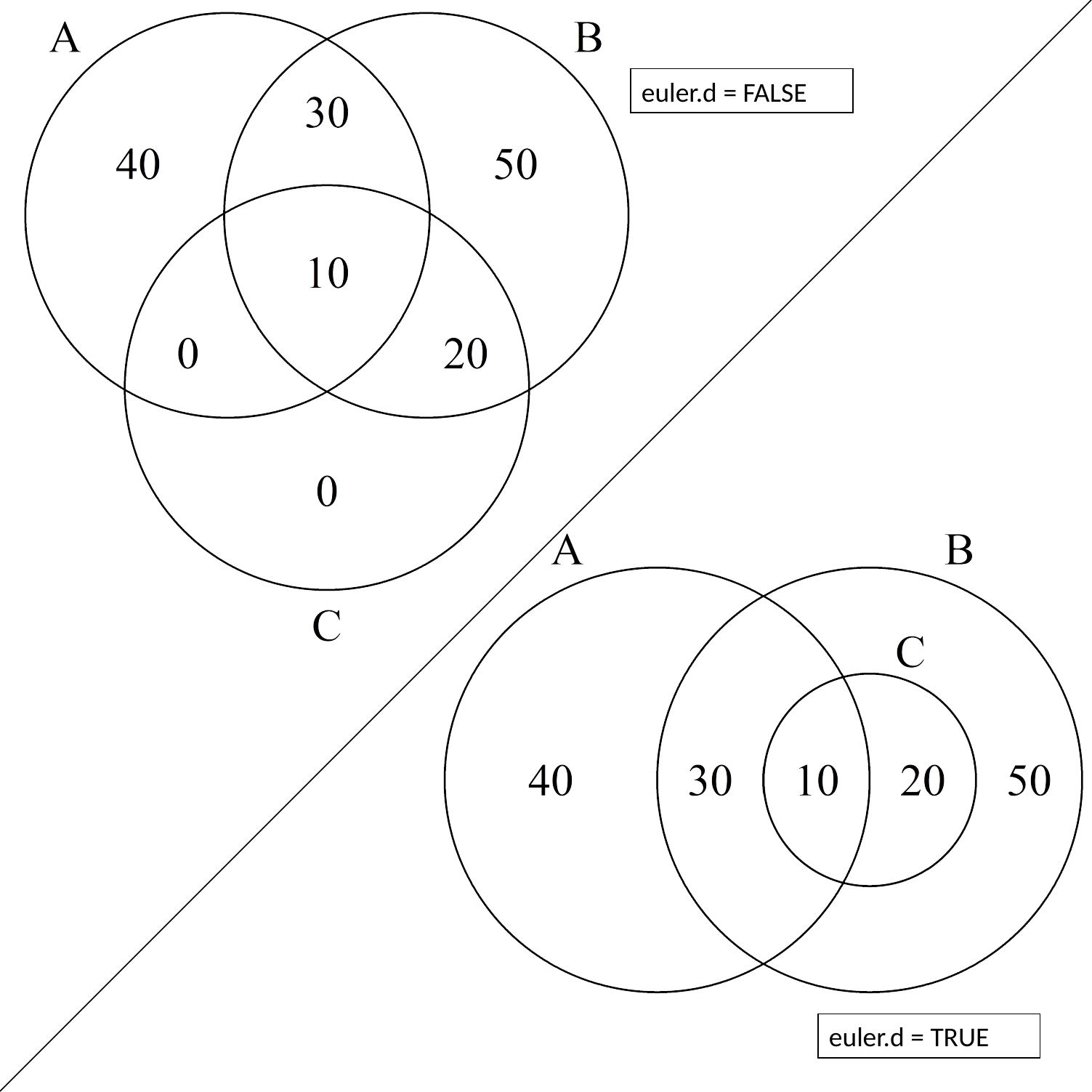

euler.d = FALSE
euler.d = TRUE

## Slide 5
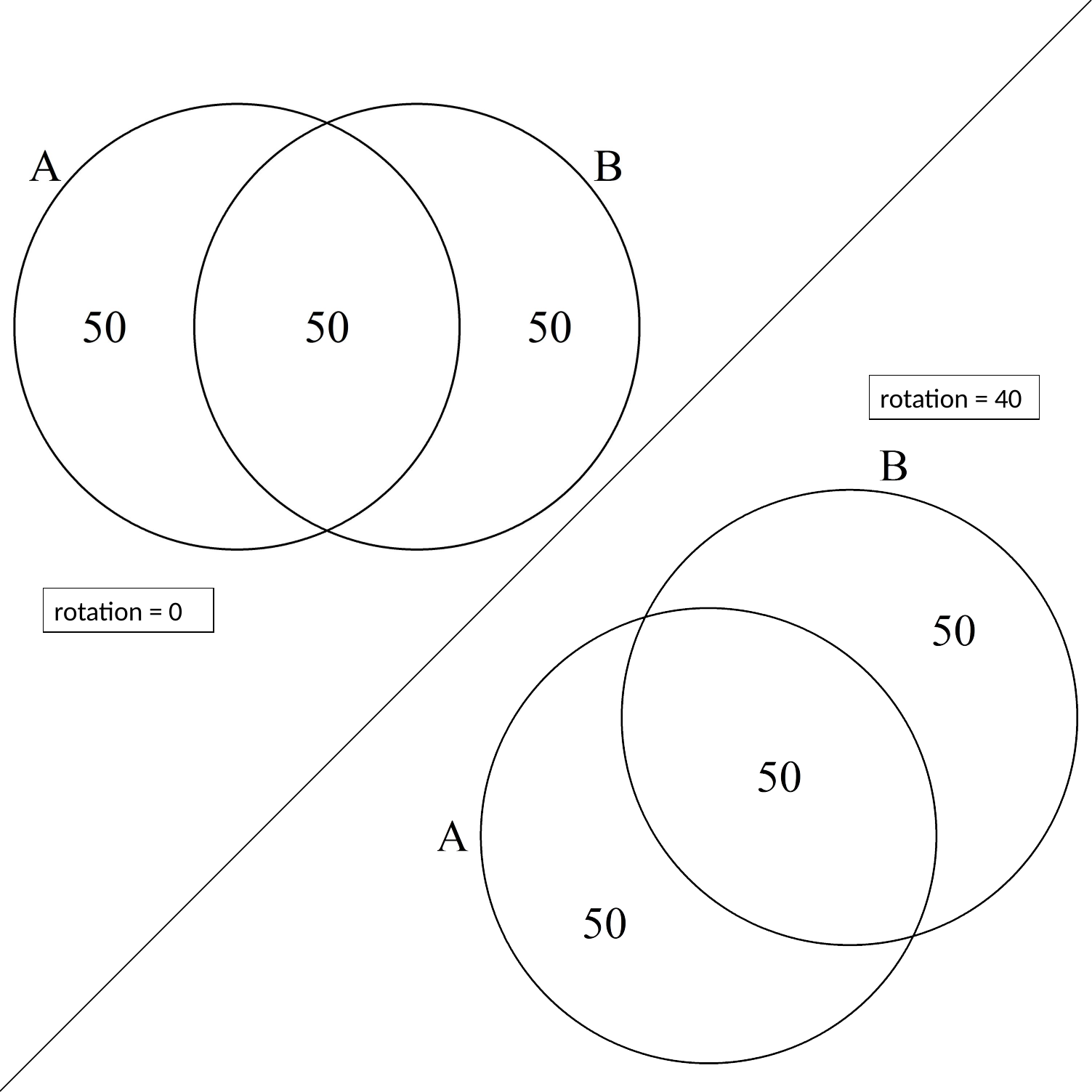

rotation = 40
rotation = 0

## Slide 6
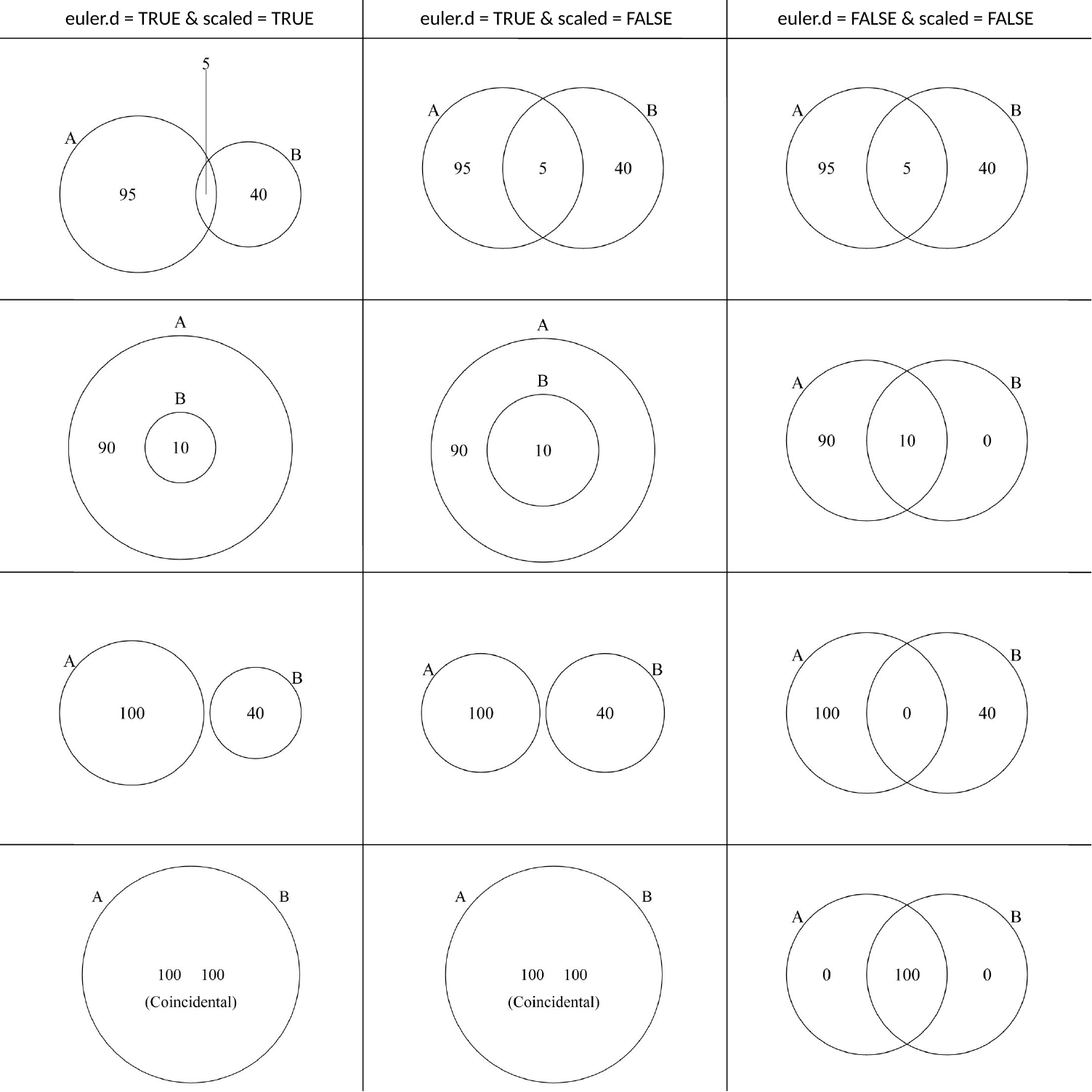

euler.d = TRUE & scaled = TRUE
euler.d = TRUE & scaled = FALSE
euler.d = FALSE & scaled = FALSE
